# Toward a conceptual framework of the acceptability of tuberculosis treatment in children using a theory generative approach

**DOI:** 10.1371/journal.pgph.0001267

**Published:** 2022-12-22

**Authors:** Dillon T. Wademan, Megan Palmer, Susan Purchase, Marieke M. van der Zalm, Muhammad Osman, Anthony J. Garcia-Prats, James A. Seddon, H. Simon Schaaf, Anneke C. Hesseling, Ria Reis, Lindsey J. Reynolds, Graeme Hoddinott

**Affiliations:** 1 Desmond Tutu TB Centre, Department of Paediatrics and Child Health, Faculty of Medicine and Health Sciences, Stellenbosch University, Cape Town, South Africa; 2 School of Human Sciences, Faculty of Education, Health and Human Sciences, University of Greenwich, London, United Kingdom; 3 Department of Paediatrics, University of Wisconsin School of Medicine and Public Health, Madison, WI, United States of America; 4 Department of Infectious Diseases, Imperial College London, London, United Kingdom; 5 Department of Anthropology, University of Amsterdam, Amsterdam, The Netherlands; 6 Department of Sociology and Social Anthropology, Faculty of Arts and Social Sciences, Stellenbosch University, Cape Town, South Africa; 7 Pivot Collective, Cape Town, South Africa; Boston University, UNITED STATES

## Abstract

To describe an early-stage holistic framework towards evaluating factors that impact the overall acceptability of TB treatment along the TB care cascade in children. We developed a conceptual framework utilising a theory generative approach. Domains were developed through review of existing definitions and analysis of existing qualitative data undertaken in acceptability studies of TB treatment in children. Clarity of domain definitions was achieved through iterative refinement among the research team. Three domains, each comprising several dimensions, were identified to holistically evaluate treatment acceptability: (1) usability, which involves the alignment between the requirements of treatment use and caregivers’ and children’s ability to integrate TB treatment into their everyday routines, (2) receptivity, which describes the end-user’s perception and expectations of treatment and its actual use, and (3) integration, which describes the relationship between available health services and caregivers/children’s capacity to make use of those services. Our framework addresses the gaps in current research which do not account for the influence of caregivers’ and children’s contexts on TB treatment uptake and overall acceptability. This approach may support the development of more standard, holistic measures to improve TB treatment delivery and experiences and future research in children.

## Background

Tuberculosis (TB) treatment for drug-susceptible (DS) and drug-resistant (DR)-TB and TB prevention in children and adolescents includes regimens comprising multiple drugs given daily, either as fixed-dose combinations or single-drug formulations. These may be challenging to prepare, administer and ingest [1, 2]. The predominant definition of treatment acceptability is “the overall ability of the patient and caregiver (defined as ‘user’) to use a medicinal product as intended (or authorised)” [3]. However, globally recognised criteria to define and standard methods to assess overall treatment acceptability have not been established [4]. Improving TB treatment acceptability for children and adolescents is increasingly recognised as a global health priority, as poor acceptability of treatment for prevention and treatment along the TB care cascade, may increase the risk of loss-to-follow-up (LTFU) and adversely affect treatment outcomes [5, 6]. Research suggests losses along the TB treatment continuum of 10.8–20.0% among children and unfavourable outcomes of 10–17% [7–11]. No research to date, describes the associations between treatment acceptability and LTFU and children’s health outcomes.

Broadly, ‘acceptability’ research has included evaluations of behaviour change interventions, health care access, marketing research, perceptions of new technologies and treatment [12–18]. Acceptability of drug treatment in children has typically been limited to assessing palatability (which includes the smell, taste, aftertaste, and mouthfeel of drugs) and ease of administration, often using adherence as a proximal indication of acceptability [19–21]. However, individual patient-related factors including co-morbidities, treatment adverse effects, and psychological responses may also impact treatment uptake and adherence thus overall acceptability [22–26]. Additional broader socio-environmental factors that may contribute to TB treatment acceptability, such as stigmatisation, social determinants of health, poverty and poor functioning health systems, have not been considered [24, 27].

Increased focus on TB in children and adolescents over the past two decades has led to breakthroughs in treatment and care [28, 29]. Among the available TB treatment formulations, the dispersible, taste-masked, fixed-dose combination (FDC) drug formulations for DS-TB regimens in children have been hailed as a marked improvement [30]. Similar development of more child-friendly formulations of second-line TB medications, such as levofloxacin, moxifloxacin, linezolid, bedaquiline and delamanid, have followed [6, 31, 32]. These child-friendly formulations are reportedly more palatable, and are therefore supposedly more acceptable to children and their caregivers [33–35]. However, much work remains to be done to design shorter, less complicated regimens for TB prevention and treatment of disease which are easier to complete, with fewer adverse effects to further improve acceptability of regimens [28, 33, 36].

In this manuscript we employed a broader conceptualisation of treatment acceptability and interrogated the acceptability of TB treatment for children along the TB care cascade including prevention and treatment of disease. The more holistic evaluation of the acceptability of TB treatment regimens and care processes could identify opportunities for intervention and improved treatment experiences, thus potentially improving outcomes. A more comprehensive understanding of the acceptability of TB treatment among children and their caregivers is especially important in high TB-burden settings, where social determinants of health influence TB risk, access to care and treatment processes [37–39]. A single framework that proposes a holistic conceptual model of TB treatment acceptability in children that includes psychosocial factors has not yet been described [4, 20, 40]. Translating and defining the many aspects of acceptability which can be practicably measured to help establish a more standardised model for the evaluation of TB treatment acceptability is challenging [41, 42]. We aim to fill this gap by proposing an initial conceptual framework to guide the evaluation of acceptability of TB treatment among children and their caregivers.

## Materials and methods

### Study design

We employed an inductive, theory generative approach to develop a conceptual framework of factors influencing TB treatment acceptability among children and their caregivers [43, 44].

### Iterative process of generating domains and dimensions for conceptual framework

We first explored existing definitions and operationalisations of acceptability, as previously described, and measured at different points along the TB care cascade (Table 1). We then looked for commonalities and unique components across those definitions and operationalisations. Thereafter we grouped and re-organised these, including suggesting superordinate domains with subordinate dimensions, through several iterations between four of the authors (DTW, RR, LJR and GH). In this process, we renamed, combined, and collapsed, some domains or dimensions to ensure greatest clarity, with consideration of mutually exclusive dimensions and definitional precision. For example, definitions of ‘accommodation’, ‘approachability’, and ‘cultural sensitivity’ overlap substantially, and conversations between co-authors involved identifying that these definitions had to do with (1) receptivity of treatment and care, and (2) health systems interactions. Additional conversations involved excluding some concepts thought to be beyond the scope of the definition of acceptability (e.g., accessibility which involves standardised measures of engagement with the health system) and introduced additional dimensions to cover perceived gaps in the conceptual model, like stigma which, though well-known to impact TB treatment experiences, has not been included in measures of acceptability.

**Table 1:** Previously described and measured dimensions of treatment acceptability.

Dimension components and definitions	Existing Dimensions of acceptability
The relationship between caregivers/children’s ability to use treatment as instructed to achieve appropriate adherence levels and health outcomes	Acceptability [3, 21, 45, 46]
The relationship between the location of healthcare services and the location of patients (including factors like transport costs, travel time and distance to facilities).	Accessibility [13, 14]
The relationship between health systems processes and patient’s perceptions of and ability to utilise health systems processes.	Accommodation [13, 14, 47]
The relationship between healthcare costs and patients’ capacity to afford these (and correlated) costs.	Affordability [13–15]
The relationship between healthcare providers’ and patients’ attitudes about personal, behavioural and health characteristics, towards one another.	Approachability [14, 47]
The relationship between the type and volume of healthcare services offered and those needed by caregivers/children.	Availability [13–15, 47]
The relationship between patients’ knowledge or understanding of available healthcare services and health workers knowledge	Awareness [14]
All health facilities, goods, and services must be culturally appropriate and sensitive to the needs of caregivers/children of different genders and ages.	Cultural sensitivity [20, 48–50]
Response to treatment regimen (including packaging and treatments’ appeal, taste, smell, mouth feel and even sound) and ability to assimilate the treatment into everyday life (including the treatment’s preparation, administration, and storage)	Dose palatability, frequency and route of administration [20, 49, 51]
Physiological response to the treatment regimen (which may be affected by age, comorbidities, or stage of development).	Patient factors [20, 48, 52]
The design factors that affect the experience and ability to utilise the pharmaceutical product as intended.	Usability [43, 53, 54]

**Fig 1:**
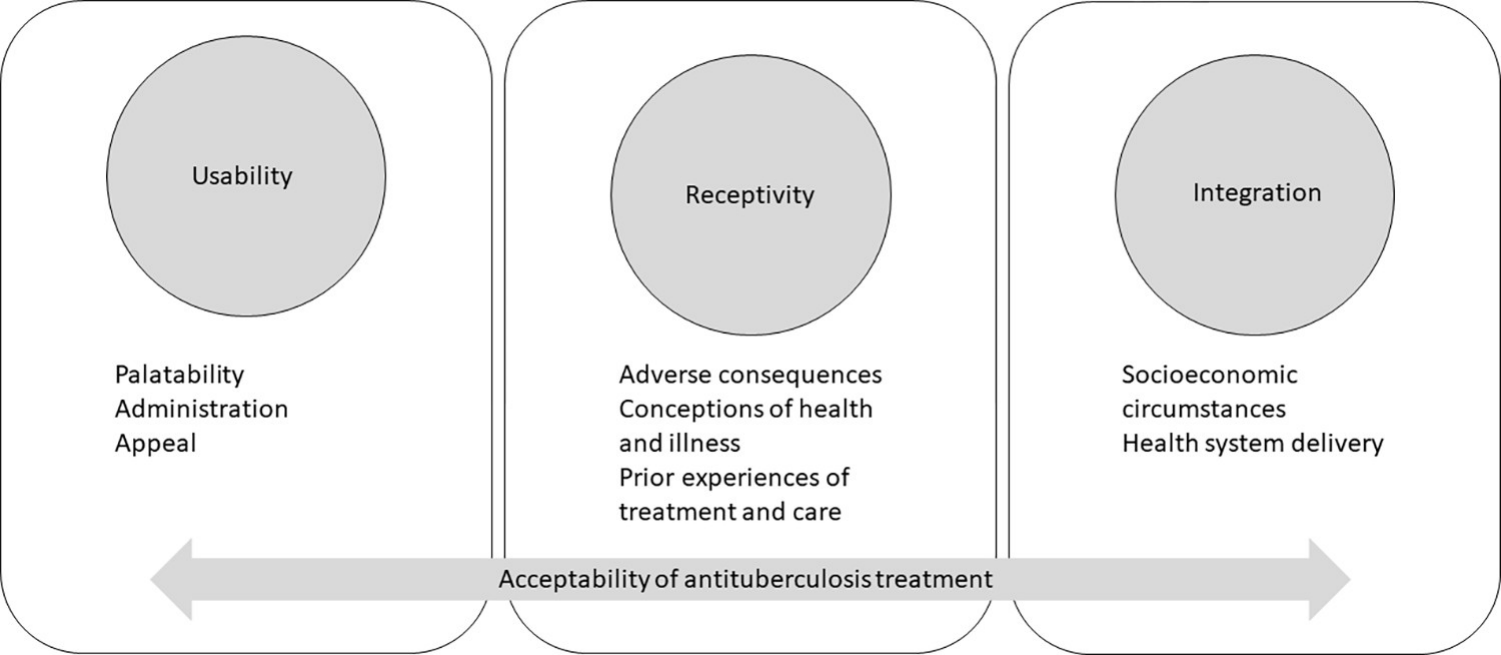
Diagrammatic depiction of conceptual framework for determining overall TB treatment acceptability.

In parallel, we explored data from three qualitative studies of the experiences of children and their caregivers on standard TB treatment regimens for prevention and treatment (see Table 2). We used those data to identify real-world, illustrative examples of the suggested dimensions which we then discussed to refine our definitional clarity and mutual understanding. We also considered whether any aspects of the children and their caregivers’ experiences of their TB treatment were being missed in our iteratively developing conceptual model and refined as needed. We shared a draft conceptual model with illustrative examples with co-authors to further refine the dimensions, domains, and ensure clarity of illustrative examples. Once we had reasonable consensus, we created a diagram to depict the conceptual model. This diagram (Fig 1) and its components were further refined through four iterations of internal author review. We present this conceptual model with illustrative examples as a preliminary framework through which acceptability of TB treatment could be evaluated in future.

**Table 2:** Semi-structured interviews with caregivers and children in three qualitative studies of treatment acceptability.

Study	Number of participants	Type of Participant		Child’s gender	Age range (months (M) years (Y))
SHINE	14		Caregiver of child	5 male: 9 female	9 M– 12 Y
TB-CHAMP	10		Caregiver of child	4 male: 6 female	9 M– 5 Y
MDR-PK2	15		Child	6 male: 9 female	2 Y– 16 Y

### Illustrative examples of TB treatment acceptability

The data are drawn from qualitative acceptability studies of three complementary TB treatment trials in children: SHINE (treatment of DS-TB disease), TB-CHAMP (prevention of drug-resistant disease), and MDR-PK2 (treatment of drug-resistant disease) [2, 56, 57]. In each, the aim was to better understand which factors contribute to, or influenced TB treatment acceptability among caregivers and children. SHINE was a randomised trial which compared the safety and efficacy of 4 versus 6 months of daily WHO-recommended FDCs of first-line TB drugs in HIV-positive and HIV-negative children with non-severe DS-TB in four countries [55]. These FDCs were intended to be child-friendly dispersible formulations that had undergone substantial taste-masking. TB-CHAMP is a randomized clinical trial comparing levofloxacin to a placebo as TB preventive therapy (TPT) for child MDR-TB contacts under 5 years of age [56]. The examples were drawn from an exploratory qualitative study within the TB-CHAMP lead-in study, during which a novel dispersible, taste-masked levofloxacin formulation was evaluated [35]. MDR-PK2 was an observational study of the pharmacokinetics and safety of MDR-TB treatment in HIV-positive and HIV-negative children routinely treated as per local standard of care for MDR-TB [2]. All data were collected as in-depth, semi-structured interviews (transcribed and translated verbatim) between September 2016 and July 2018. Separate acceptability studies for SHINE, TB-CHAMP, and MDRPK2 have been reported elsewhere [33–35]. The diversity in clinical research samples (prevention, treatment, drug-resistant, drug susceptible, observational and interventional), provided a broad context through which to establish a conceptual understanding of TB treatment acceptability among children and caregivers.

### Ethics and consent

All acceptability evaluations, including this study (S20/08/204), were approved by the Health Research Ethics Committee, Stellenbosch University (SHINE M14/09/044; TB-CHAMP M16/02/009; MDR-PK2 N15/02/012). All participants (aged ≥18) provided written informed consent, and children (aged 7–17) provided written informed assent, with formal written consent obtained from the child’s parent/legal guardian. All methods were carried out in accordance with relevant guidelines and regulations.

## Findings

We suggest a novel conceptualisation of overall treatment acceptability, informed by Tables 1 and 3, comprising three broad domains and eight dimensions within these domains (Fig 1). Domain one (usability) refers to the properties of the TB treatment itself and includes dimensions of palatability, administration processes, and appeal. Domain two (receptivity) is the alignment between the treatment and end-users’ perceptions and includes dimensions on adverse consequences, conceptions of health and illness, and prior experiences of treatment and care. Domain three (integration) is the relationship between the treatment and its implementation in the systems/environments where it is intended to be used and includes dimensions of socioeconomic circumstances and health system delivery. Below, we describe each domain and dimension in turn, and in Table 3 provide further illustrative examples using participant quotes from the three qualitative sub-studies.

**Table 3:** Quotes from each qualitative sub-study, relevant to each domain and its correlating dimensions.

Domain of TB treatment acceptability	Category	Study	Quote
Usability	Palatability (taste, smell, feel)	SHINE	“No, she doesn’t make an ugly face, but she cries […] she cries because its ugly because she doesn’t like things that are bitter” (20161013, Caregiver, 8M, female)
		TB-CHAMP	“I was not happy at all with those pills because it is, oh it’s extremely bitter. [. . .] I would never drink those pills. […] The new medication that they have given her, the Levofloxacin is much better” (20170131, Caregiver, 5Y, female).
		MDR-PK2	“It tastes joh! [gosh!] [it] doesn’t taste right, it tastes bad, very bad! […] For me it’s better to drink it with the water, and if I finished drinking [the tablets] then I eat a small stick of sweet, just to take that sour away” (20180706, 16Y, male).
	Ease of use	SHINE	“It’s very easy […] it’s better than the normal tablets. Yes, because if they gave normal tablets to him then he’s not going to take it […]. I pour a little water in the cup, and I gave it to him like that […] if there is still residue then I throw a little water in again, and gave it to him and let him drink it by himself” (20161006, Caregiver, 5Y, male)
		TB-CHAMP	“The one pill is ground and then I mix [the pill] in eight mil (8ml) of water, then I only pull up three mils (3ml) [in a syringe] [. . .] then the [rest] must be thrown away (20180803, Caregiver, 5Y, female).
		MDR-PK2	“Okay like you can take two here with these ones […] here you take two [of these tablets] as well, and here two […] here there are six […] yes and one here. […] it is [sixteen] in all […] I eat all of them at once” (20180705, 16Y, male).
	Appeal	SHINE	“Yes, its bitter […] but now, since it’s orange I thought it was like *Drink ‘o Pop* [orange flavoured juice] but when I tasted it, just little, I can’t taste the [juice] taste” (20160926, Caregiver, 6Y, female).
		TB-CHAMP	“You’ll even get her mocking you when she says it, ‘pills, pills, pills,’ she doesn’t like it. […] A syrup, when you shake it, she likes it. Maybe it could be a syrup, this treatment for children could be better. [The colour could be] like red, you know have you seen a child with something that is red? It’s like it’s nice […] like those medications that are red and glisten? Like she comes with her mouth open, she likes nice things” (20170316, Caregiver, 3Y, female).
		MDR-PK2	“I think it made it scarier, like if you take a big box […]. Like I know when you have HIV and AIDS, there’s like a [big] box for them […]. It makes me scared. Like what’s in there, a thousand million tablets? […] All the other tablet boxes are very plain white, and they have no interesting label […]. So, I’m not gonna take that. […] If I designed a box [I would make it] very colourful, like creative, and then [children] would be like ‘that looks interesting, I think I want to see what it is’ and they would like it” (20180702, 14Y, female)
Receptivity	Adverse (physiological) consequences	SHINE	“If she has had something to eat then there isn’t a problem, but if she doesn’t have anything in her stomach then it almost looks like it makes her nauseous […] it she makes her nauseous actually […] because she doesn’t have anything in her stomach” (20161018, Caregiver, 2Y, female).
		TB-CHAMP	“Since I’ve put him on those pills now, his, here by his genitals and his buttocks, it comes out as a rash, red. I want to know now, maybe can it be [the pills] that’s causing it or what, because [the other pills] I gave him didn’t have that effect” (20170315, Caregiver, 7M, male).
		MDR-PK2	“I don’t feel better when I get home, I just feel like vomiting, I feel nauseous. […] I just can’t do work like I should at home, like my mom taught me to […] to make dresses, do work, cooking. Now it’s hard to do it because I have [DR-]TB” (20180628, 14Y, female).
	Adverse (psychosocial) consequences	SHINE	“He doesn’t want to go to school now because he’s having these [glands], so he’s shy” (20160908, Caregiver, 8Y, male).
		TB-CHAMP	“Sometimes I feel overwhelmed and ask why do I have TB? [. . .] I had a friend. I watched her die. [. . .] Sometimes I question, I ask myself why, why TB? And sometimes I feel insane. Why, when I am around other people, do I have to wear a mask? It’s not nice” (20170306, Caregiver, 6M, male).
		MDR-PK2	“I found that I am also not okay, because I was also sick [with DR-TB]. I thought about my child, how people may find her disgusting because they didn’t even want her to sit next to her [while she was in hospital]. She couldn’t even leave [her room] or sit in the passage” (20180710, Caregiver, 13Y, female).
	Conceptions of health and illness	SHINE	“I was having an old lady there she was keeping her until she was one year six months. So, I think they were smoking too much, the grandmother and the grandfather. I suspect maybe she got it there, but I am not sure, so I don’t want to say” (20160919, Caregiver, 2Y, female).
		TB-CHAMP	“Every day you must administer the tablets. [. . .] As long as she gets it in, because if you take poison [. . .] it’s like a poison, if you put it in her, it will affect her. It doesn’t matter how, or how much of the pill you put in it will somehow affect her” (20170222, Caregiver, 2Y, female).
		MDR-PK2	“the brother had a hookah pipe that they smoke like that. I think he picked it up smoking together” (20180712, Caregiver, 12Y, male).
	Prior experiences of treatment and care	SHINE	“I went to the pharmacy to get medication, but the medication would run out and there’s no change. Then I thought., let me go to the clinic so they can also give me pills. But then the pills here at the clinic weren’t working. […] Then I said he must go produce sputum at the clinic. When his tests came back, they were negative. […]. Then when we went in and got the x-ray, it came back saying positive.” (20160929, Caregiver, 10Y, male).
		TB-CHAMP	“In 2005 I had TB […] it was painful because you can’t eat the pills, they are a lot and when you eat them, they are bitter […] mine were six [tablets] every day. […] I got diagnosed [with HIV] in 2005, at the same time. […] Yes, when I found out I hated myself and I didn’t want to accept it” (20170320, Caregiver, 9M, female).
		MDR-PK2	“She got TB when we lived with my mother […]. My brother died from TB because he didn’t take his medication. […] My twins, they recovered from MDR-TB, but when she, my baby got it, it was more difficult! […] I felt terrible when the clinic phoned to tell me she has MDR-TB.” (20180702, Caregiver, 14Y, female).
User-health system interface	Socio-economic circumstances	SHINE	“If she spits up that thing [the treatment], it’s difficult to wash out her clothes […] it’s red! It’s difficult. […] No, it’s not easy [to get out of the clothes]. […] Sometimes I have to take off all her clothes and then must give her [the pills]” (20160915, Caregiver, 3Y, female).
		TB-CHAMP	“So now instead of giving me injections five times a week […] I get injections three times a week. Yes, [I must] travel there to get the injection [three times a week]. See! That’s a lot of money, and I don’t even work” (20170124, Caregiver, 2Y, triplets).
		MDR-PK2	“He was still at school. He was supposed to finish matric and then before the exam, he became so sick. […] On the morning that he had to write his exams, his legs gave in, so he couldn’t [write his exams]. So, I took him to the doctor, the private doctor. The doctor said […] the x-ray shows it is [DR-]TB. […] So, he didn’t finish [his exams]” (20180712, Caregiver, 12Y, male).
	Health system delivery	SHINE	“We took him [to our local clinic] […] I didn’t like the service of the nurse because they didn’t even touch him, he was just listening to what I was saying. So, I said ‘okay let me take him to another clinic in the afternoon.’ […]. They only gave us the results after when we were at the [District Hospital]” (20160912, Caregiver, 9M, male).
		TB-CHAMP	“The person [living with DR-TB] is our neighbour. […] The health workers didn’t call me. They said it was good that we came because our neighbour has TB […]. They write us a letter to go to [the district hospital] for the test and for the X-ray, […] the results came back negative. But they said, ‘we want to be sure,’ so they wrote us a letter again to send us to [the tertiary hospital] and they do the tests and x-rays again, and the results come back negative. So, they sent us back to the clinic to have the prevention of the TB” (20170314, Caregiver, 4Y, female)
		MDR-PK2	“It was not nice. It was painful because they weren’t even staying with me. It wasn’t nice for my daughter either, because she would always cry when I left her here [at the specialised hospital], so it was not nice […] she stayed at [the specialised hospital] for eight months” (20180725, Caregiver, 3Y, female).

### Domain 1: User-drug interface or ‘usability’

Usability is the alignment between the requirements of daily administration and caregivers’ and children’s ability to incorporate TB treatment into their daily routine. Challenges in usability interfere with integrating TB treatment into everyday routine. We suggest that usability includes treatment palatability, administration considerations, and appeal.

#### Palatability

Palatability describes children’s responses to the physical characteristics of the TB treatment (defined as smell, taste, aftertaste and mouth feel, and sight and sound) [4]. For example, a sixteen-year-old adolescent boy said the treatment tastes horrible, making him gag. He tried to neutralise the poor palatability by ingesting the treatment with a lot of water, immediately followed by eating something sweet. Poor palatability negatively impacts usability, and therefore overall acceptability. Additionally, when children dislike the taste of the treatment, it adds to caregivers’ burden of care as they must overcome their children’s resistance to ensure treatment adherence. The caregiver of an eight-month-old girl said the bitter taste of treatment amplified her daughter’s resistance to treatment administration. The caregiver had to improvise new ways to administer treatment to her daughter every day, adding to the emotional and physical labour of care.

#### Administration

Administration is defined as how easily caregivers or children (if self-administering patients) can open, prepare, and ingest their treatment. Longer periods to prepare and administer or ingest the prescribed dosages because formulations are not user-friendly negatively affects usability. A caregiver of a six-year-old girl on TPT for MDR-TB said she would have to crush one pill in 8mls of water and draw out 3 mils to administer to her child. TB treatment that requires crushing pills, measuring, and administering fractions of dosages to children is especially time consuming and onerous for caregivers. Additionally, drug-food interactions are important to consider, since many caregivers use foods, sweets, or liquids, simultaneously to drug administration or to console children after administration. The increased burden of care on caregivers created by lengthy and complicated administration processes negatively impacts treatment acceptability by reducing its usability. Integrating TB treatment administration into everyday activities is complicated when caregivers are given rigid time intervals in which to administer treatment. Additionally, the number of pills at each administration and the frequency of dosing can hinder integration into everyday household routine.

#### Appeal

Appeal is used to describe the size and colour of drug formulations, packaging and the clarity of accompanying treatment messaging or instructions. Formulations that are an unappealing colour or are difficult to store/handle may deter children from treatment uptake and adherence. In one case, a fourteen-year-old girl compared brown pills to oil and said they could not be pleasant. Similarly, treatment packaging colours, instructions and messaging should be appealing, clear and intelligible to the population responsible for treatment administration. It also indicates the importance of ensuring treatment processes are described in the appropriate language suitable for the child and caregivers’ educational level and understanding of TB disease and treatment.

If treatment instructions or the messaging on treatment packaging is too complicated, it may be difficult for users to share responsibility for the administration of and adherence to TB treatment with others. One caregiver said she could not trust her husband to administer treatment to their three-year-old daughter because he had not attended the clinic with her and the instructions on the treatment packaging were too complex for him to follow. This negatively impacted the caregivers’ ability to integrate her child’s TB treatment into everyday routines in the family context and therefore its overall acceptability.

### Domain 2: User-treatment interface ‘receptivity’

Receptivity refers to the association between end users’ (caregivers’ and children’s) expectations about treatment, and the actual experience of treatment. For example, if the end users hold a health belief that the worse a drug tastes the more effective it must be, then child-friendly formulations of TB treatment that have high palatability may still have poor acceptability because of a poor match to expectations. We suggest three dimensions important to this relationship: (1) balance between TB prevention or treatment benefits against adverse consequences, (2) coherence with conceptions of health and illness, and (3) coherence with prior experiences of TB treatment and other treatments.

#### Adverse consequences

Adverse consequences include any detrimental physiological and/or psychosocial consequences children and/or caregivers experience following their child’s treatment initiation. Adverse physiological consequences of TB treatment (including but not limited to, nausea and vomiting, pain, itching and changes in skin colour or texture) negatively impact treatment acceptability. For example, a fourteen-year-old girl on treatment for MDR-TB reported feeling disoriented and lethargic after ingesting treatment, disrupting her ability to contribute to household activities and normal everyday functioning. Her physiological inability to contribute to household chores, also negatively impacted her psychosocial wellbeing. Disruptions to caregivers’ and children’s normal social functioning negatively impacted their experience of TB treatment and therefore its acceptability.

Other adverse psychosocial consequences of TB disease and treatment include withdrawal of social and financial support, isolation, interpersonal conflict, depression, and stigmatisation. The caregiver of a thirteen-year-old-girl on MDR-TB treatment, described how she felt guilty for exposing her daughter (and others) to MDR-TB. Family members and health workers subsequently treated her and her child with apprehension. The internal psychological strain of exposing her child to MDR-TB was exacerbated by others’ negative behaviour towards her and her child. The experience of feeling ostracised by people in their social network adversely affects TB treatment acceptability, as people may be deterred from returning to the clinic and/or adhering to treatment.

#### Conceptions of health and illness

Conceptions of health and illness are defined as caregivers’/children’s knowledge and expectations of TB transmission, disease, and treatment. Incongruences between caregivers’ and children’s conceptions of health and illness–including their perceptions of the efficacy of treatment–can negatively impact acceptability. For example, the caregiver of a two-year-old girl compared TPT for MDR-TB to poison, believing any amount of treatment is sufficient to prevent her child from developing TB disease. The caregivers’ misunderstanding of how treatment works resulted in underdosing the child, negatively impacting her health outcomes. Misconceptions of underlying treatment mechanisms may delay healthcare access, treatment uptake and adherence, or even cure and therefore overall acceptability.

#### Prior and current experiences of treatment and care

Prior experiences of treatment include experiences of TB treatment and other treatment experiences that may negatively or positively influence caregivers’ and children’s willingness to engage with TB treatment. Caregivers with prior experience of TB disease may access care earlier for their children, as they are more aware of the signs and symptoms of TB and are better able to navigate the health system’s processes to secure a diagnosis. The caregiver of a ten-year-old-boy said she helped enable her child’s diagnosis by identifying signs and symptoms and navigating the health system’s processes after having had TB herself.

Children and caregivers with negative prior experiences of TB treatment (or other treatment) may be less willing and able to administer treatment to their children. For instance, the caregiver of a nine-month-old-boy living with HIV said her son immediately knew when she was going to administer his TB treatment or antiretroviral treatment (ART) and cried during every administration episode. Her child’s experience of ART biased his acceptance of TB treatment. Concurrently administering TB treatment alongside other chronic medications can add to the burden of care and negatively impact overall acceptability among children and caregivers. Conversely, being in long term care and having experienced adherence support services either for a chronic illness (e.g., for HIV) or prior TB episode may also improve understanding and uptake.

### Domain 3: User-health system interface ‘integration’

We use user-health system interface to describe the degree of fit between the health systems delivery of TB treatment and care, and the end user’s capacity to utilise that care. We suggest that TB treatment-health system interface, or ‘integration,’ includes socio-economic circumstances and health systems processes like level of care offered, as well as the accessibility and availability of TB treatment related services.

#### Socio-economic circumstances

Caregivers’ socio-economic circumstances may hinder their ability to access health services or may impact TB treatment adherence. For instance, a caregiver of a six-year-old girl said her child’s appetite dramatically increased after starting TB treatment and providing sufficient food became unaffordable. As a result, she stopped her child’s treatment. Although this is likely a greater reflection on generalised poverty, it also suggests that TB treatment is impeded when families are not properly supported, and their financial security is threatened. A sixteen-year-old adolescent boy with MDR-TB said he had been set back two academic school years after being hospitalised for MDR-TB. The long-term social and financial consequences of not graduating may deter caregivers and children from seeking and adhering to TB treatment.

#### Health system delivery

Health system delivery includes all those processes involved in securing a timely and accurate diagnosis, receiving the appropriate TB treatment, and ensuring continuity of care until treatment completion. Diagnostic and health systems delays may add to caregivers’ psychological distress, as well as financial, time, and physical burden of care. For example, the caregiver of a nine-month-old boy said she had to visit two clinics and a district hospital before her child received a TB diagnosis. The caregiver complained about the additional financial and time-related costs of care incurred even before starting TB treatment. Diagnostic or treatment delays potentially undermine caregivers’ and children’s confidence in the health system and treatment.

Additional health system processes that incur psychosocial and financial costs, including hospitalisation, may affect health care seeking behaviour. The caregiver of a three-year-old girl with DR-TB who received her treatment as an in-patient complained about the costs involved in regularly visiting her child. She also said she and her child experienced separation anxiety every time she visited and left her daughter at the hospital. Health systems processes that incur excessive financial, social, or emotional costs, or which delay treatment initiation may negatively impact caregivers’ and children’s experience of TB treatment and overall acceptability.

## Discussion

We initially highlight the incongruencies between the broad definition of acceptability, “the overall ability of the patient and caregiver (defined as ‘user’) to use a medicinal product as intended (or authorised)” [3], and existing measures of acceptability of TB treatment in children which primarily focus on palatability and ease of use, particularly among children. We therefore attempted to develop a more holistic conceptual model of acceptability of TB treatment among children and caregivers. Following a theory-generative and iterative process, we propose three domains that encompasses most factors relevant to the overall acceptability of TB treatment: usability, receptivity, and integration. Usability encompasses the alignment between the characteristics most immediately related to the preparation, administration, and use of TB treatment, including the ability to incorporate TB treatment into daily routine. Receptivity involves the relationship between end users’ expectations of the TB treatment and the lived experience of taking TB treatment. Integration involves the association between the health system’s delivery of TB treatment and the end user’s capacity to access and make use of the TB treatment within their context.

Other research on the acceptability of a digital health technology intervention for diagnosing and treating childhood pneumonia in resource-limited settings included understanding the impact of patients’ and caregivers’ economic condition and perceptions of the device’s efficacy [53]. Sekhon et al., argue that the concept of ‘acceptability’ remains “ill-defined, under-theorized, and poorly assessed” [57]. Among the recent conceptual frameworks of treatment acceptability, however, none have been developed that focus on TB treatment in children [4]. Our conceptual model builds on other work that has advocated for drug developers, health services and healthcare workers to collaborate and respond to the myriad pragmatic, financial and social determinations that caregivers and children experience during their TB treatment journeys [33, 50, 58, 59]. Similar to others, we found that the domains/dimensions may overlap [13]. Furthermore, challenges experienced in one domain or dimension may have implications for other domains/dimensions. For example, stigmatisation which falls within the receptivity domain is linked to the appeal of treatment packaging which falls within our ‘usability’ domain. The interrelatedness of each domain resembles the biopsychosocial health model which describes how biological, psychological, and social systems impact individuals’ health possibilities and outcomes [60, 61]. More research is needed to better explore and understand how the domains and correlating dimensions of TB treatment acceptability intersect with and/or influence one another.

Strengths of our theory-generative process include the grounding of our suggested domains within data from three studies with caregivers and children across a wide age spectrum, with and without HIV, and receiving different treatment regimens for prevention and treatment. Additionally, the data were drawn from a diverse group of people from different cultural and ethnic backgrounds. Importantly we included both children and caregivers’ perceptions and experiences of TB treatment in this study. Furthermore, we used multiple iterations of engagement with the published literature and an interdisciplinary team of authors to ensure coherence and applied relevance. Limitations for extrapolation of our conceptual model include that it is based on an inductive process rather than on empirical research. The illustrative examples were all from South Africa, although the clinical context varied substantially, and acceptability may differ by context. More research must be done to determine whether the acceptability of treatment from health workers’ perspectives may influence treatment management processes. Furthermore, this conceptual model requires further empirical research to determine its utility and applicability in different settings including in routine care. Additional investigation of each conceptual domain and related dimensions is necessary to generate a standardised and itemised scale to measure overall acceptability among children and caregivers. Although our framework is informed by the acceptability of TB treatment in children and their caregivers in South Africa, it may have application across different age groups and settings.

Our proposed conceptual framework presents an opportunity to identify key obstacles within households, communities, and healthcare systems to optimise the degree of fit between patients’ needs and available treatment for children with TB. It provides the first steps towards a global standard against which novel treatment strategies could be measured to determine overall TB treatment acceptability. Previously, research on the acceptability of TB treatment in children was scattered and unsystematic. This framework focuses future research on TB treatment acceptability by providing three defined and described domains which can be investigated collectively or separately. Furthermore, this conceptual framework provides a common language through which the acceptability of treatment regimens, strategies, and related health system processes can be studied and compared. Lastly, the framework provides the field with a model with which to determine the acceptability of novel TB treatment strategies in children in the context of family-centred care.

## References

[1] Graham SM, Grzemska M, Gie RP. The background and rationale for a new fixed-dose combination for first-line treatment of tuberculosis in children. Int J Tuberc Lung Dis. 2015;19(12): 3–8. doi: 10.5588/ijtld.15.0416 26564534

[2] Garcia-Prats AJ, Svensson EM, Weld ED, Schaaf HS, Hesseling AC. Current status of pharmacokinetic and safety studies of multidrug-resistant tuberculosis treatment in children. Int J Tuberc Lung Dis. 2018;22(5): 15–23. doi: 10.5588/ijtld.17.0355 29665949

[3] Kozarewicz P. Regulatory perspectives on acceptability testing of dosage forms in children. Int J Pharm. 2014;469(2): 245–248. doi: 10.1016/j.ijpharm.2014.03.057 24704104

[4] Mistry P, Batchelor H. Evidence of acceptability of oral paediatric medicines: a review. J Pharm Pharmacol. 2017;69(4): 361–376. doi: 10.1111/jphp.12610 27524471

[5] Snow KJ, Cruz AT, Seddon JA, Ferrand RA, Chiang SS, Hughes JA, et al. Adolescent tuberculosis. Lancet Child Adolesc Heal. 2020;4(1): 68–79. Available from: doi: 10.1016/S2352-4642(19)30337-2 31753806 PMC7291359

[6] WHO. Consolidated Guidelines on Tuberculosis Treatment. Module 5: Management of tuberculosis in children and adolescents. Geneva: Switzerland: World Health Organisation Publications; 2022. 99 p. 35404556

[7] Chenciner L, Annerstedt KS, Pescarini JM, Wingfield T. Social and health factors associated with unfavourable treatment outcome in adolescents and young adults with tuberculosis in Brazil: a national retrospective cohort study. Lancet Glob Heal. 2021;9(10):e1380–1390. 10.1016/S2214-109X(21)00300-4 34534486

[8] Chiang SS, Starke JR, Miller AC, Cruz AT, Del Castillo H, Valdivia WJ, et al. Baseline predictors of treatment outcomes in children with multidrug-resistant tuberculosis: a retrospective cohort study. Clin Infect Dis. 2016;63(8):1063–1071. doi: 10.1093/cid/ciw489 27458026

[9] Enane LA, Lowenthal ED, Arscott-Mills T, Matlhare M, Smallcomb LS, Kgwaadira B, et al. Loss to follow-up among adolescents with tuberculosis in Gaborone, Botswana. Int J Tuberc Lung Dis. 2016;20(10): 1320–1325. doi: 10.5588/ijtld.16.0060 27725042

[10] Kibirige L, Izudi J, Okoboi S. Discontinuation of tuberculosis treatment among children in the Kampala Capital City Authority health facilities: a mixed-methods study. BMC Infect Dis. 2021;21(1): 1–10. 10.1186/s12879-021-06244-y 34074268 PMC8167996

[11] Osman M, Meehan SA, von Delft A, Du Preez K, Dunbar R, Marx FM, et al. Early mortality in tuberculosis patients initially lost to follow up following diagnosis in provincial hospitals and primary health care facilities in Western Cape, South Africa. PLoS One. 2021;16(6 June):1–15. doi: 10.1371/journal.pone.0252084 34125843 PMC8202951

[12] Kazdin AE. Acceptability of child treatment techniques: The influence of treatment efficacy and adverse side effects. Behav Ther. 1981;12(4): 493–506. 10.1016/S0005-7894(81)80087-1

[13] Penchansky R, Thomas JW. The concept of access: Definition and relationship to consumer satisfaction. Med Care. 1981;19(2): 127–140. doi: 10.1097/00005650-198102000-00001 7206846

[14] Saurman E. Improving access: Modifying Penchansky and Thomas’s theory of access. J Heal Serv Res Policy. 2016;21(1):36–39. doi: 10.1177/1355819615600001 26377728

[15] McIntyre D, Thiede M, Birch S. Access as a policy-relevant concept in low- and middle-income countries. Heal Econ Policy Law. 2009;4(2): 179–193. doi: 10.1017/S1744133109004836 19187569

[16] Ha YP, Tesfalul MA, Littman-Quinn R, Antwi C, Green RS, Mapila TO, et al. Evaluation of a Mobile Health Approach to Tuberculosis Contact Tracing in Botswana. J Health Commun. 2016;21(10): 1115–1121. doi: 10.1080/10810730.2016.1222035 27668973 PMC6238947

[17] Wiklund ME, Kendler J, Yale AS. Usability testing of medical devices. New York: CRC Press, Taylor & Francis Group; 2011. 393 p.

[18] Carter SL. Further conceptualisation of treatment acceptability. Educ Train Dev Disabil. 2008;43(2): 135–143. https://www.jstor.org/stable/23879925

[19] Walsh J, Ranmal SR, Ernest TB, Liu F. Patient acceptability, safety and access: A balancing act for selecting age-appropriate oral dosage forms for paediatric and geriatric populations. Int J Pharm. 2018;536(2): 547–562. doi: 10.1016/j.ijpharm.2017.07.017 28705619

[20] Rieder M. Size and taste matters: recent progress in the development of age-appropriate medicines for children. Pharmaceut Med. 2018;32(1): 21–30. 10.1007/s40290-017-0218-2

[21] Ruiz F, Vallet T, Pensé-Lhéritier A-M, Aoussat A. Standardized method to assess medicines’ acceptability: focus on paediatric population. J Pharm Pharmacol. 2017;69(4):406–16. doi: 10.1111/jphp.12547 27109018 PMC5396311

[22] Stillson CH, Okatch H, Frasso R, Mazhani L, David T, Matlhare M, et al. ‘That’s when I struggle’… Exploring challenges faced by care givers of children with tuberculosis in Botswana. Int J Tuberc Lung Dis. 2016;20(10):1314–9. 10.5588/ijtld.15.0989 27725041

[23] Dudley L, Mukinda F, Dyers R, Marais F, Dagmar S. Mind the gap! Risk factors for poor continuity of care of TB patients discharged from a hospital in the Western Cape, South Africa. PLoS One. 2018;13(1):e0190258. doi: 10.1371/journal.pone.0190258 29370162 PMC5784914

[24] Franck C, Seddon JA, Hesseling AC, Schaaf HS, Skinner D, Reynolds L. Assessing the impact of multidrug-resistant tuberculosis in children: an exploratory qualitative study. BMC Infect Dis. 2014;14(1):1–10. doi: 10.1186/1471-2334-14-426 25084990 PMC4127187

[25] Loveday M, Sunkari B, Master I, Daftary A, Mehlomakulu V, Hlangu S, et al. Household context and psychosocial impact of childhood multidrug-resistant tuberculosis in KwaZulu-Natal, South Africa. Int J Tuberc Lung Dis. 2018;22(1):40–6. doi: 10.5588/ijtld.17.0371 29297424

[26] Dawson LA. What factors affect adherence to medicines? Arch Dis Child Educ Pract Ed. 2019;104(1):49–52. doi: 10.1136/archdischild-2017-312820 29374624

[27] Skinner D, Claassens M. It’s complicated: why do tuberculosis patients not initiate or stay adherent to treatment? a qualitative study from South Africa. BMC Infect Dis. 2016;16(1):1–9. doi: 10.1186/s12879-016-2054-5 27887646 PMC5124309

[28] Marais BJ. Newer drugs for tuberculosis prevention and treatment in children. Indian J Pediatr. 2019;86(8):725–31. doi: 10.1007/s12098-018-02854-8 30707347

[29] Marais BJ, Schaaf HS. Childhood tuberculosis: an emerging and previously neglected problem. Infect Dis Clin North Am. 2010;24(3):727–49. doi: 10.1016/j.idc.2010.04.004 20674801

[30] WHO. New fixed-dose combinations for the treatment of TB in children. Towards Zero Deaths. Geneva; 2016.

[31] The Sentinel Project for Pediatric Drug-Resistant Tuberculosis. Pediatric formulations of second-line drugs for the treatment of drug-resistant tuberculosis. Boston, USA: Sentinel Project on Pediatric drug-resistant tuberculosis and the Global Drug Facility; 2018. 1–6 p. Available from: http://sentinel-project.org/

[32] The Sentinel Project for Pediatric Drug-Resistant Tuberculosis. Management of Drug-Resistant Tuberculosis in Children: A Field Guide. 4th ed. Boston, USA: Sentinel Project on Pediatric drug-resistant tuberculosis; 2018. 77 p. Available from: http://sentinel-project.org/

[33] Wademan DT, Busakwe L, Nicholson TJ, van der Zalm M, Palmer M, Workman J, et al. Acceptability of a first-line antituberculosis formulation for children: qualitative data from the SHINE trial. Int J Tuberc Lung Dis. 2019 Dec 1;23(12):1263–8. doi: 10.5588/ijtld.19.0115 31931909 PMC6903808

[34] Winckler J, Draper HR, Schaaf SH, Hesseling AC, Garcia-Prats AJ. Acceptability of levofloxacin, moxifloxacin and linezolid among children and adolescents treated for TB. Int J Tuberc Lung Dis. 2020;24(12):1316–8. doi: 10.5588/ijtld.20.0544 33317680 PMC8320765

[35] Purchase SE, Garcia-Prats AJ, De Koker P, Draper HR, Osman M, Seddon JA, et al. Acceptability of a novel levofloxacin dispersible tablet formulation in young children exposed to multidrug-resistant tuberculosis. Pediatr Infect Dis J. 2019;38(6):608–10. doi: 10.1097/INF.0000000000002268 30550511

[36] Chiang SS, Roche S, Contreras C, Del Castillo H, Canales P, Jimenez J, et al. Barriers to the treatment of childhood tuberculous infection and tuberculosis disease: a qualitative study. Int J Tuberc Lung Dis. 2017;21(2):154–60. http://www.ncbi.nlm.nih.gov/pubmed/28234078 doi: 10.5588/ijtld.16.0624 28234078

[37] Lönnroth K, Jaramillo E, Williams BG, Dye C, Raviglione M. Drivers of tuberculosis epidemics: The role of risk factors and social determinants. Soc Sci Med. 2009;68(12):2240–6. doi: 10.1016/j.socscimed.2009.03.041 19394122

[38] Thomas BE, Shanmugam P, Malaisamy M, Ovung S, Suresh C, Subbaraman R, et al. Psycho-socio-economic issues challenging multidrug resistant tuberculosis patients: a systematic review. PLoS One. 2016;11(1):e0147397. doi: 10.1371/journal.pone.0147397 26807933 PMC4726571

[39] Yates TA, Ayles H, Leacy FP, Schaap A, Boccia D, Beyers N, et al. Socio-economic gradients in prevalent tuberculosis in Zambia and the Western Cape of South Africa. Trop Med Int Heal. 2018;23(4):375–90. doi: 10.1111/tmi.13038 29432669 PMC6022780

[40] Ranmal SR, O’Brien F, Lopez F, Ruiz F, Orlu M, Tuleu C, et al. Methodologies for assessing the acceptability of oral formulations among children and older adults: a systematic review. Drug Discov Today. 2018;23(4):830–47. doi: 10.1016/j.drudis.2018.01.038 29371123

[41] Sam T, Ernest TB, Walsh J, Williams JL. A benefit/risk approach towards selecting appropriate pharmaceutical dosage forms—An application for paediatric dosage form selection. Int J Pharm. 2012;435(2):115–23. doi: 10.1016/j.ijpharm.2012.05.024 22626885

[42] Drumond N, van Riet-Nales DA, Karapinar-Çarkit F, Stegemann S. Patients’ appropriateness, acceptability, usability and preferences for pharmaceutical preparations: results from a literature review on clinical evidence. Int J Pharm. 2017;521(1–2):294–305. doi: 10.1016/j.ijpharm.2017.02.029 28229945

[43] Oliver C. Critical realist grounded theory: A new approach for social work research. Br J Soc Work. 2012;42(2):371–87.

[44] Timmermans S, Tavory I. Theory construction in qualitative research: from grounded theory to abductive analysis. Sociol Theory. 2012;30(3):167–86.

[45] EMA. Guidelines on pharmaceutical development of medicines for paediatric use [Internet]. European Medicines Agency: Science, Medicines & Health. London: European Medicines Agency; 2013. 24 p. Available from: https://www.ema.europa.eu/en/documents/scientific-guideline/guideline-pharmaceutical-development-medicines-paediatric-use_en.pdf

[46] Carter SL. Review of recent treatment acceptability research. Educ Train Dev Disabil. 2007;42(3):301–16.

[47] Levesque J-F, Harris M, Russell G. Patient-centred access to health care: conceptaulising access at the interface of health systems and populations. Int J Equity Health. 2013;12(18):1–9. 23496984 10.1186/1475-9276-12-18 PMC3610159

[48] Liu F, Ranmal S, Batchelor HK, Orlu-Gul M, Ernest TB, Thomas IW, et al. Patient-centred pharmaceutical design to improve acceptability of medicines: similarities and differences in paediatric and geriatric populations. Drugs. 2014;74(16):1871–89. doi: 10.1007/s40265-014-0297-2 25274536 PMC4210646

[49] Boffa J, Mayan M, Ndlovu S, Mhlaba T, Williamson T, Sauve R, et al. The role of agency in the implementation of Isoniazid Preventive Therapy (IPT): Lessons from oMakoti in uMgungundlovu District, South Africa. PLoS One. 2018;13(3):1–16. doi: 10.1371/journal.pone.0193571 29513719 PMC5841771

[50] Bryson SP. Patient-centred, administration friendly medicines for children—An evaluation of children’s preferences and how they impact medication adherence. Int J Pharm. 2014;469(2):257–9. doi: 10.1016/j.ijpharm.2014.04.069 24797763

[51] Ternik R, Liu F, Bartlett JA, Khong YM, Thiam Tan DC, Dixit T, et al. Assessment of swallowability and palatability of oral dosage forms in children: Report from an M-CERSI pediatric formulation workshop. Int J Pharm. 2018;536(2):570–81. doi: 10.1016/j.ijpharm.2017.08.088 28844897

[52] WHO. Toolkit for research and development of paediatric antiretroviral drugs and formulations [Internet]. Geneva: World Health Organisation Press; 2018. 84–109 p. Available from: https://www.who.int/hiv/pub/5.pdf

[53] Ginsburg AS, Agyemang CT, Ambler G, Delarosa J, Brunette W, Levari S, et al. MPneumonia, an innovation for diagnosing and treating childhood pneumonia in low-resource settings: a feasibility, usability and acceptability study in Ghana. PLoS One. 2016;11(10):1–14. 10.1371/journal.pone.0165201 PMC5082847 27788179

[54] Rosenbaum S. Usability evaluations versus usability testing: when and why? IEEE Trans Prof Commun. 1989;32(4):210–6.

[55] Turkova A, Wills GH, Wobudeya E, Chabala C, Palmer M, Kinikar A, et al. Shorter treatment for nonsevere tuberculosis in African and Indian children. N Engl J Med. 2022;386(10):911–22. doi: 10.1056/NEJMoa2104535 35263517 PMC7612496

[56] Seddon JA, Garcia-prats AJ, Purchase SE, Osman M, Demers A, Hoddinott G, et al. Levofloxacin versus placebo for the prevention of tuberculosis disease in child contacts of multidrug-resistant tuberculosis: study protocol for a phase III cluster randomised controlled trial (TB-CHAMP). Trials. 2018;19(693):1–11. doi: 10.1186/s13063-018-3070-0 30572905 PMC6302301

[57] Sekhon M, Cartwright M, Francis JJ. Acceptability of health care interventions: A theoretical framework and proposed research agenda. Br J Health Psychol. 2018;23(3):519–31. 29453791 10.1111/bjhp.12295

[58] Hodes R, Vale B, Toska E, Cluver LD, Dowse R, Ashorn M. ‘Yummy or Crummy: the multisensory components of medicines-taking among HIV-positive youth.’ Glob Public Health. 2018;14(2):284–99. doi: 10.1080/17441692.2018.1504103 30067457

[59] Zimri K, Casper R, Hoddinott G, Schaaf HS, Garcia-Prats AJ, Rose P, et al. A novel approach for eliciting adolescent MDR-TB treatment tolerability: qualitative data from South Africa. Int J Tuberc Lung Dis. 2020;24(1):43–7. doi: 10.5588/ijtld.19.0207 32005306

[60] Engel G. The need for a new medical model: a challenge for biomedicine. Science (80-). 1977;196(4286):129–36. 10.1126/science.847460 847460

[61] Karunamuni N, Imayama I, Goonetilleke D. Pathways to well-being: Untangling the causal relationships among biopsychosocial variables. Soc Sci Med. 2021;272(February 2020):112846. doi: 10.1016/j.socscimed.2020.112846 32089388

